# Disseminated* Mycobacterium chimaera* Presenting as Vertebral Osteomyelitis

**DOI:** 10.1155/2017/9893743

**Published:** 2017-04-24

**Authors:** Daphne M. Moutsoglou, Frank Merritt, Ethan Cumbler

**Affiliations:** Department of Medicine, Hospital Medicine Group, University of Colorado School of Medicine, Aurora, CO, USA

## Abstract

*Mycobacterium chimaera*, a member of the* Mycobacterium avium *complex, is a slow-growing, nontuberculous mycobacterium associated with outbreaks in cardiac-surgery patients supported on heart-lung machines. We report a case of an elderly woman on chronic prednisone who presented with a six-month history of worsening chronic back pain, recurrent low-grade fevers, and weight loss. Imaging identified multilevel vertebral osteomyelitis and lumbar soft-tissue abscess. Abscess culture identified* M. chimaera*.

## 1. Introduction

*Mycobacterium chimaera *is a slow-growing, nontuberculous mycobacterium (NTM) sequevar belonging to the* Mycobacterium avium *complex (MAC) [1].* M. chimaera *has been reported in patients undergoing cardiac surgery, who later develop cardiac and disseminated infection [2–4].* M. chimaera *can contaminate tap water and has been identified growing in water tanks within heater-cooler units of heart-lung machines [5–7].

Prior reports show that, despite antibiotic and surgical treatment, disseminated* M. chimaera *has a high mortality rate. Even with antibiotic therapy targeted to* in vitro *sensitivities, medical therapy often fails to result in clearance. The identification of* M. chimaera *may be missed in the absence of molecular sequencing diagnostics. While this case represents a description of proven* M. chimaera *osteomyelitis, it is likely that other cases have occurred and been attributed to other MAC species.

## 2. Case

A 75-year-old woman with systemic lupus erythematosus was evaluated for two months of chronic, low back pain. She also reported subjective fevers, weight loss, and an initially necrotic, one-centimeter wound on her right calf that developed five months earlier. She had no history of trauma, paraspinal steroid injections, or past cardiac or spine surgery. Medications are significant for prednisone and hydroxychloroquine.

On exam she was afebrile, with normal vitals, mild lumbar tenderness, normal strength, and intact reflexes in the lower extremities and without saddle anesthesia.

Laboratory analysis showed normocytic anemia (hemoglobin 11.6 g/dL), normal white blood cells (5.4 × 10^9^/L), normal platelets (175 × 10^9^/L), hyponatremia (124 mmol/L), and elevated erythrocyte sedimentation rate (106 mm/hr) and C-reactive protein (117 mg/L). She had normal serum complement levels, a negative HIV, and an indeterminate QuantiFERON test.

**Figure 1:**
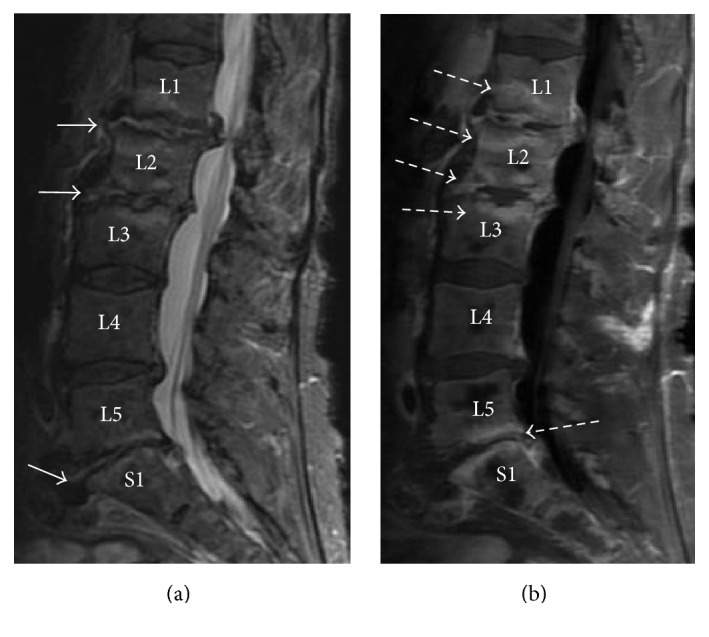
*Prevertebral osteomyelitis, discitis, and soft-tissue abscess seen on sagittal MRI imaging with and without contrast*. (a) Sagittal T2 STIR showing high signal consistent with fluid in the L1-2, L2-3, and L5-S1 disc spaces with endplate edema (white arrows). (b) Sagittal T1 fat saturation obtained after contrast shows extensive enhancement centered around the L1–3 and L5-S1 disc spaces consistent with discitis and osteomyelitis (dashed arrows).

MRI with and without contrast of the thoracic and lumbar spine revealed multilevel discitis and prevertebral osteomyelitis at L1-2, L2-3, and L5-S1 (Figure 1). An abscess located in L5-S1 (Figure 1(a), white arrow) was aspirated. Culture of the abscess revealed polymorphonuclear cells without organisms on conventional and acid-fast bacilli staining. Bacterial and fungal microscopy of the abscess and blood cultures were obtained. Cryptococcal PCR,* Coccidioides* antibody, and* Mycobacterium tuberculosis *polymerase chain reaction were negative.

The patient was started on empiric intravenous vancomycin and ertapenem. On day 12,* M. chimaera *was identified in the abscess aspirate by culture and sequencing. She was then transitioned to clarithromycin, rifampin, and ethambutol. A new biopsy of the calf wound was positive for* M. chimaera.* Surgical discectomy, debridement, and fusion were recommended; however, she deferred surgery and was discharged with a prolonged course of antimycobacterials.

## 3. Discussion

Disseminated infection with* M. chimaera *has been previously described following cardiac surgery (range of 5 to 40 months to diagnosis after surgery) as vascular graft infection, prosthetic valve endocarditis, or myocarditis [2, 3]. Outbreaks of* M. chimaera *in patients supported by heart-lung machines have prompted recent investigations identifying water tanks supporting heater-cooler systems as the likely source of infection in these outbreaks [6, 7].* M. chimaera *can also cause pulmonary infection, similar to* M. avium *and* M. intracellulare* [8–11]. Initial reports suggested that* M. chimaera *is more virulent than the other MAC species [1]. This has since been called into question by a larger case series in which* M. chimaera *was more likely to be a colonizer of the respiratory tract and less likely to cause true infection, compared to the other MAC species [11]. In this series, patients with true respiratory infection due to* M. chimaera *were more likely to be immunosuppressed (53%), suggesting a more opportunistic pattern of infection [11].

Similar to other MAC species,* M. chimaera *is typically treated with a prolonged course of clarithromycin, ethambutol, and rifampin. Vertebral osteomyelitis caused by MAC or other NTM often requires surgery, due to either neurologic deficits, spinal instability, or failure of medical treatment [12].

Identification of* M. chimaera *requires sequencing; therefore, nonsequencing methods may fail to identify the correct* Mycobacterium *species [3, 11, 13]. A study that retrospectively sequenced samples from patients with diagnosed MAC found that 28% of infections were due to* M. chimaera*, while 54% and 18% of previously diagnosed MAC infections were due to* M. avium *and* M. intracellulare*, respectively [11]. In another retrospective study of patients with prior diagnosis of* M. intracellulare*, sequencing determined that 143 of the 166 samples attributed to* M. intracellulare *infection could be reclassified as* M. chimaera *[13]. Similar to other MAC infections,* M. chimaera *often presents with fever of unknown origin and weight loss.

Disseminated* M. chimaera* infection after cardiac surgery may manifest as granulomatous nephritis, granulomatous hepatitis, chorioretinitis, multifocal choroiditis, and pulmonary infection, as well as spondylodiscitis, osteoarthritis, and poststernotomy wound infection [1–4]. Laboratory characteristics of disseminated* M. chimaera *include an elevated C-reactive protein, pan-negative conventional blood cultures, with anemia, lymphopenia, and thrombocytopenia, and elevated lactate dehydrogenase, creatinine, and transaminases [2–4].

The patient in this case presented with a history of chronic nonhealing leg wound months prior to the clinical signs of her vertebral osteomyelitis. Given that the leg wound later grew acid-fast bacilli identified as* M. chimaera*, her ulcer was likely the first manifestation of the disease, months before diagnosis of spinal involvement. This emphasizes the importance of considering slow-growing NTM species in the evaluation of nonhealing wounds in patients on immune-compromising medications, since conventional pathology and bacterial cultures will fail to make the diagnosis.

Unlike many prior descriptions of patients with* M. chimaera*, our patient did not have a history of recent surgery or heart-lung bypass. Her chronic immunosuppression with prednisone and hydroxychloroquine was a likely predisposing factor to this infection. She had an elevated CRP and anemia similar to other reports, but she did not have pancytopenia or liver and kidney involvement, as described in other patients with disseminated disease after heart surgery [2–4]. To our knowledge, this is the first report in the literature of* M. chimaera *causing vertebral osteomyelitis in a patient without history of cardiac surgery.

## Abbreviations

MAC: * Mycobacterium avium* complex
NTM: Nontuberculous mycobacteria.

## References

[1] Tortoli E., Rindi L., Garcia M. J. (2004). Proposal to elevate the genetic variant MAC-A, included in the *Mycobacterium avium* complex, to species rank as *Mycobacterium chimaera* sp. nov. *International Journal of Systematic and Evolutionary Microbiology*.

[2] Kohler P., Kuster S. P., Bloemberg G. (2015). Healthcare-associated prosthetic heart valve, aortic vascular graft, and disseminated *Mycobacterium chimaera* infections subsequent to open heart surgery. *European Heart Journal*.

[3] Sax H., Bloemberg G., Hasse B. (2015). Prolonged outbreak of *Mycobacterium chimaera* infection after open-chest heart surgery. *Clinical Infectious Diseases*.

[4] Achermann Y., Rössle M., Hoffmann M. (2013). Prosthetic valve endocarditis and bloodstream infection due to *Mycobacterium chimaera*. *Journal of Clinical Microbiology*.

[5] Wallace R. J., Iakhiaeva E., Williams M. D. (2013). Absence of *Mycobacterium intracellulare* and presence of *Mycobacterium chimaera* in household water and biofilm samples of patients in the United States with *Mycobacterium avium* complex respiratory disease. *Journal of Clinical Microbiology*.

[6] Sommerstein R., Rüegg C., Kohler P., Bloemberg G., Kuster S. P., Sax H. (2016). Transmission of *Mycobacterium chimaera* from heater-cooler units during cardiac surgery despite an ultraclean air ventilation system. *Emerging Infectious Diseases*.

[7] Haller S., Höller C., Jacobshagen A. (2016). Contamination during production of heater-cooler units by *Mycobacterium chimaera* potential cause for invasive cardiovascular infections: results of an outbreak investigation in Germany, April 2015 to February 2016. *Eurosurveillance*.

[8] Cohen-Bacrie S., David M., Stremler N., Dubus J.-C., Rolain J.-M., Drancourt M. (2011). *Mycobacterium chimaera* pulmonary infection complicating cystic fibrosis: a case report. *Journal of Medical Case Reports*.

[9] Alhanna J., Purucker M., Steppert C. (2012). *Mycobacterium chimaera* causes tuberculosis-like infection in a male patient with anorexia nervosa. *International Journal of Eating Disorders*.

[10] Bills N. D., Hinrichs S. H., Aden T. A., Wickert R. S., Iwen P. C. (2009). Molecular identification of *Mycobacterium chimaera* as a cause of infection in a patient with chronic obstructive pulmonary disease. *Diagnostic Microbiology and Infectious Disease*.

[11] Boyle D. P., Zembower T. R., Reddy S., Qi C. (2015). Comparison of clinical features, virulence, and relapse among *Mycobacterium avium* complex species. *American Journal of Respiratory and Critical Care Medicine*.

[12] Kim C.-J., Kim U.-J., Kim H. B. (2016). Vertebral osteomyelitis caused by non-tuberculous mycobacteria: predisposing conditions and clinical characteristics of six cases and a review of 63 cases in the literature. *Infectious Diseases*.

[13] Schweickert B., Goldenberg O., Richter E. (2008). Occurrence and clinical relevance of *Mycobacterium chimaera* sp. nov., Germany. *Emerging Infectious Diseases*.

